# Candidate imaging biomarkers for *PMP22* ‐related inherited neuropathies

**DOI:** 10.1002/acn3.51561

**Published:** 2022-06-03

**Authors:** Alison R. Roth, Jun Li, Richard D. Dortch

**Affiliations:** ^1^ Division of Neuroimaging Research Barrow Neurological Institute Phoenix Arizona USA; ^2^ Department of Neurology Vanderbilt University Nashville Tennessee USA; ^3^ Department of Neurology Wayne State University Detroit Michigan USA; ^4^ Vanderbilt University Institute of Imaging Science Vanderbilt University Nashville Tennessee USA; ^5^ Department of Radiology and Radiological Sciences Vanderbilt University Nashville Tennessee USA; ^6^ Department of Biomedical Engineering Vanderbilt University Nashville Tennessee USA

## Abstract

**Objective:**

Charcot–Marie–Tooth type 1A (CMT1A) and hereditary neuropathy with liability to pressure palsy (HNPP) are caused by mutations to the *peripheral myelin protein 22* (*PMP22*) gene. A need exists for sensitive and reliable biomarkers of progression and treatment response. Magnetic resonance imaging (MRI) metrics of nerve pathology and morphology were investigated for this purpose.

**Methods:**

MRI was performed at 3.0 T in the thigh of CMT1A (*N* = 11) and HNPP patients (*N* = 12) and controls (*N* = 23). Three potential imaging biomarkers of the sciatic nerve were investigated: 1) magnetization transfer ratio (MTR), which assays myelin content, and 2) cross‐sectional area (CSA) and 3) circularity, which assay morphological changes. Potential imaging biomarkers were compared across cohorts and assessed for relationships with disability in the legs (CMTES_L_), compound motor action potentials (CMAP), and motor conduction velocities (MCV). Inter‐rater reliability and test–retest repeatability were established for each imaging metric.

**Results:**

Significant differences in MTR, CSA, and circularity were observed in CMT1A relative to controls (*p* = 0.02, *p* < 0.001, and *p* = 0.003, respectively, via Wilcoxon rank‐sum tests). Differences were not observed in the HNPP cohort. Significant relationships were observed between MTR and clinical metrics (CMTES_L_: *p* = 0.003, CMAP: *p* = 0.03, MCV: *p* = 0.01); and between CSA and electrophysiology (CMAP: *p* = 0.002, MCV: *p* < 0.001). All metrics were reliable and repeatable with MTR the most reliable (intraclass correlation coefficient [ICC] >0.999, CV = 0.30%) and repeatable (ICC = 0.84, CV = 3.16%).

**Interpretation:**

MTR, CSA, and circularity showed promise as reliable and sensitive biomarkers of CMT1A, but not HNPP. These warrant longitudinal investigation as response biomarkers in upcoming clinical trials of CMT1A, while other methods should be considered for HNPP.

## Introduction

*Peripheral myelin protein 22* (*PMP22*) is a transmembrane glycoprotein expressed primarily in myelinating Schwann cells after birth. As such, it plays a significant role in myelin formation and maintenance, although it has also been implicated in cell survival, proliferation, and death.^1^ While the role of *PMP22* in myelination is not fully understood, recent work suggests that *PMP22* plays a role in the formation of myelin tight/adherens junctions that seal the spaces between myelin lamina.^2^ Furthermore, duplications and deletions of the *PMP22* gene cause two autosomal dominant inherited neuropathies: Charcot–Marie–Tooth disease type 1A (CMT1A) and hereditary neuropathy with liability to pressure palsy (HNPP).^1^

CMT1A is caused by a 1.4 Mb duplication in chromosome 17p11.2 that includes the *PMP22* gene,^3^, ^4^ which results in a slowly progressive, symmetrical, dysmyelinating polyneuropathy with secondary length‐dependent (or “dying back”) axonal loss.^5^, ^6^ Clinically, CMT1A is characterized by distal weakness, sensory loss, foot deformities, absent reflexes, and uniformly slowed nerve motor conduction velocities (MCV).^7^, ^8^ HNPP results from a heterozygous deletion or loss of function of the same gene that encodes for *PMP22*.^1^ Initial symptoms of HNPP, by contrast, are transient and often occur in response to mild mechanical stress such as compression, stretching, or repetitive motions of the symptomatic limb that would be innocuous to a healthy person. Clinically, HNPP is characterized by localized numbness, weakness, and paralysis. Nerve conduction studies (NCS) show slowed MCV at sites susceptible to mechanical pressure regardless of symptoms at the site.^9^ Pathologically, the hallmark feature of HNPP is the formation of tomaculae, or excessive myelin folding.^10^ Secondary length‐dependent axonal degeneration has also been observed.^9^ Although life expectancy is normal in both CMT1A and HNPP patients, these symptoms can significantly affect quality of life.

The CMT neuropathy score (CMTNS)^11^ is a reliable and valid biomarker of disability in inherited neuropathies. The score is based upon evaluations of sensory and motor symptoms in limbs, pin and vibration sensitivity, limb muscle strength, and NCS in the wrist. The CMTNS has been employed to measure progression and/or treatment response in natural history studies^12^, ^13^ and clinical trials.^14^, ^15^, ^16^ Unfortunately, these studies revealed that CMTNS exhibits a relatively low responsiveness longitudinally; therefore, the use of CMTNS as an outcome measurement in clinical trials would result in infeasibly large sample sizes to show treatment efficacy in CMT1A.^17^ Thus, there is a need for improved monitoring biomarkers of disease progression as outcome measures in future human trials.

Magnetic resonance imaging (MRI) provides measurements that report on muscle atrophy/fat replacement,^9^, ^18^ myelin content in nerve,^9^, ^19^ and nerve hypertrophy,^20^ each of which have been proposed as potential biomarkers of inherited neuropathies. Previous studies have shown that fat replacement on MRI correlated strongly with manual muscle testing in CMT patients^18^ and correlated moderately with disability (CMTNS) in HNPP patients.^9^ Unfortunately, fat replacement occurs downstream to nerve pathology and represents the chronic endpoint of the disease, making it potentially difficult to evaluate therapies that slow or halt progression. To overcome this, researchers have proposed direct measures of nerve pathology and morphology, including nerve magnetization transfer ratio (MTR) and nerve diameter. MTR primarily provides information on myelin content changes and relates to disability across inherited neuropathies (CMT1A, CMT2A, and HNPP),^19^ although a similar relationship was not observed in a subsequent small cohort of HNPP patients.^9^ Nerve hypertrophy is a feature of CMT1A due to the buildup of debris from chronic dysmyelinating processes, which results in the “onion bulb” appearance pathologically and has been suggested as a biomarker specific to that subtype.^21^ While significant differences in nerve size between CMT1A patients and healthy control subjects have been observed when measured on MRI, relationships between nerve hypertrophy and disability have not been assessed.^19^, ^20^ However, these relationships have been evaluated using ultrasound‐based measures of nerve size, where nerve hypertrophy in the distal nerves of CMT1A patients related to disability in both adult and pediatric populations.^22^, ^23^

This study seeks to systematically evaluate both microstructural (myelin content via MTR) and macrostructural features (nerve cross‐sectional area, or CSA, and shape) of the sciatic nerve within a cohort of patients with *PMP22*‐related diseases (CMT1A and HNPP) and healthy control subjects. Imaging metrics were considered for their ability to detect differences between the two *PMP22*‐related neuropathies and healthy control subjects. In addition, suitability to act as monitoring biomarkers in patients was evaluated via correlations to disability, inter‐rater reliability, and test–retest repeatability in a small cohort with test–retest data. Based on previous studies,^22^, ^23^ we investigated more proximal nerves in the thigh (sciatic nerve) rather than more distally to minimize floor‐effects from severely degenerated nerves. The purpose of the present study is to develop monitoring biomarkers for their potential use in future clinical trials.

## Methods

### Standard protocol approvals, registrations, and patient consents

The local Institutional Review Board approved this study, and signed consent was obtained prior to all examinations.

### Human subjects and clinical information

The CMT1A and HNPP patients in this study were recruited from the Vanderbilt University CMT Clinic, which is part of the international CMT Consortium. CMT1A and HNPP were confirmed via genetic testing in all cases. Patients with a history of diabetes, renal failure, HIV infection, or other conditions linked to peripheral neuropathies were excluded. Healthy control subjects were recruited to approximately match patients for age, sex, and body mass index (BMI). In the control group, subjects self‐reported no symptoms that were suggestive of peripheral neuropathies, which was confirmed via physical examination. Patient and control subject clinical data can be found in Table 1.

**Table 1:** Demographic and clinical data for each cohort.

	Control (*n* = 23)	CMT1A (*n* = 11)	HNPP (*n* = 12)
Male, %	39.1%	45.5%	25.0%
Age, y	42.6 ± 13.1	46.2 ± 12.7	53.0 ± 8.7
	(21.1, 60.0)	(27.9, 69.5)	(36.5, 64.8)
BMI, kg/m^2^	26.8 ± 5.5	25.3 ± 5.7	28.6 ± 5.2
	(16.8, 39.3)	(18.6, 39.6)	(19.6, 39.1)
CMTES_L_	0	8.1 ± 4.6	5.5 ± 3.5
		(1, 14)	(2, 13)
	*N* = 1	*N* = 10	*N* = 8
MCV^1^, m/s	–	4.5 ± 2.9	8.6 ± 2.0
		(0.1, 8.6)	(4.0, 11.1)
		*N* = 10	*N* = 10
CMAP^1^, mV	–	24.2 ± 5.2	46.5 ± 7.1
		(14.8, 32.0)	(34.1, 54.5)
		*N* = 10	*N* = 10

Data collected in all subjects within each cohort unless otherwise stated and presented as mean ± SD (range). BMI, body mass index; CMAP, compound motor action potential; CMT1A, Charcot–Marie–Tooth type 1A; CMTES_L_, clinical CMT evaluation score from the legs; HNPP, hereditary neuropathy with liability to pressure palsies; MCV, motor conduction velocity.

^1^ From median or ulnar nerve (or mean value when data from both nerves available).

Clinical disability was assessed via the CMTNS version 2 in all patients, and the portion of the clinical evaluations taken from the legs (CMTES_L_ range 0–20) was extracted for comparison to our MRI findings.^24^ Motor NCS data were also acquired from the median and/or ulnar nerve of a majority of patients (when available) using conventional methods.^25^ NCS was conducted in the arms because NCS are often nonresponsive in the legs of CMT patients.^26^, ^27^ The recording electrodes were placed over the thenar and hypo‐thenar muscles in the hand, reference electrodes were placed over the tendons in the wrist, and stimulation was performed at the wrist and elbow. Due to well‐established normative values in normal controls, NCS was not collected in control subjects.^28^ The mean MCV and compound motor action potential (CMAP) are reported in Table 1.

### MRI data acquisition

All subjects were scanned on a 3.0 T Philips Ingenia MR scanner (Philips Healthcare, Best, The Netherlands) equipped with a multi‐element coil for lower extremity imaging. MTR data were collected from a transverse volume in one thigh to visualize the sciatic nerve using a 3D multishot EPI sequences acquired with and without an MT saturation pulse (25‐msec pulse width, 1000° nominal flip angle, and 1.5 kHz off‐resonance).^19^, ^29^ The imaging location was standardized to center at the distal third of the femur as previously described.^19^ Parameters for this scan included: acquired/reconstructed resolution = 0.8 × 0.8 × 6 mm^3^/0.75 × 0.75 × 3 mm^3^, field‐of‐view = 192 × 192 × 144 mm^3^, TR/TE/excitation flip angle = 60 msec/11 msec/10°, water‐selective excitation pulse (to minimize fat signal), *k*‐space lines per shot = 5, SENSE factor = 1, and signal acquisitions averaged = 2. Sciatic nerve morphology was estimated from the same image volumes as described in the following section.

### Image analysis

All analysis was performed in MATLAB (Mathworks, Natick, MA) except where noted. The sciatic nerve was manually identified in the 40 central slices of the reference volume (i.e., scan without MT‐weighting). Raters were blinded to patient disease status and demographic variables during this step to minimize bias. Following this manual step, background voxels and voxels effected by partial volume averaging with fat were automatically eliminated using the MIPAV (NIH, Bethesda, MD) fuzzy c‐means segmentation algorithm, which ensured that only fascicular regions were included in each analysis.

Three imaging features were extracted from each slice and then averaged over all slices. MTR was calculated as:

$$\mathrm{MTR}=\left(1-\frac{S_{\mathrm{MT}}}{S_{\mathrm{REF}}}\right)\times 100,$$

where, $S_{\mathrm{MT}}$ and $S_{\mathrm{REF}}$ are the signal intensities from the MT‐weighted and reference images, respectively, per Dortch et al.^19^ CSA of the nerve at each slice was calculated as the number of voxels multiplied by the CSA of each voxel after correcting for the angulation of nerve relative to the imaging slice. Our previous studies noted that nerves were less cylindrical in CMT1A patients compared to the healthy control subjects. As a result, cross‐sectional circularity was calculated for each slice according to the regionprops function in MATLAB with a unit dilation of the original ROI to fill spaces between fascicles (imfill). In the case that multiple ROIs were present per slice (i.e., where the sciatic nerve bifurcates into the tibial and common peroneal nerves in the distal slices of some subjects), the circularity of each ROI was averaged for the slice. The median circularity was calculated across slices to minimize the impact of outlier values due to small ROIs.

### Repeatability

Seven control subjects were scanned a second time to evaluate the test–retest repeatability of each potential imaging biomarkers. The median time between test–retest scans in control subjects was 157 days (range 7–189 days). This allowed us to capture sources of variability over a long time period, as would be required in longitudinal studies in CMT1A/HNPP due to their slowly progressive nature.

### Statistics

Statistical analyses were performed using R software version 4.0.2 (The R Foundation, http://www.r‐project.org/). The significance level selected was *p* = 0.05. In all cases, raw *p*‐values are reported (without correcting for the effect of multiple comparisons) due to the exploratory nature of this work.

For each potential imaging biomarker, significant variations across the patient (CMT1A and HNPP) and control cohorts were evaluated using the Kruskal–Wallis test followed by post hoc pairwise Wilcoxon rank‐sum tests. The potential imaging biomarkers were then assessed for relationships with CMTES_L_ using Spearman correlation at the first scan. The relationships between demographic information and imaging metrics were assessed using partial correlations (age, BMI) or Wilcoxon rank‐sum tests (sex) in the control cohort. A multiple linear regression was also calculated to model CMTES_L_ based on a patient's demographics and imaging features. Only demographic variables found to have significant relationship with the selected imaging features (MTR, CSA) were included in the model. MTR and CSA were selected because previous literature has demonstrated a relationship with disability.^19^, ^22^, ^23^ Circularity was excluded due to its exploratory nature and to avoid issues related to collinearities between the shape‐based metrics (CSA and circularity).

Inter‐rater reliability was determined for each potential imaging biomarker based on measurements taken by a second reader in *N* = 13 patients and control subjects. Reliability was evaluated using intraclass correlation coefficient (ICC), coefficient of variation (CV), and paired *t*‐tests between MRI estimates at each scan. Finally, the inter‐scan repeatability of each potential imaging biomarker was assessed using the ICC and CV. The relative limits of agreement were calculated according to Bland–Altman analysis for repeated measurements.^30^, ^31^

## Results

### Clinical features of patients

All CMT1A patients presented with a typical history of chronic sensory loss, distal muscle weakness/atrophy, and foot deformities. HNPP patients reported a least one episode of transient, asymmetric, focal sensory loss, and weakness sometimes following mechanical stresses. NCS results showed slowed conduction velocities and decreased amplitudes in patients with CMT1A relative to patients with HNPP (Table 1).

### General features of sciatic nerve MRI

**Figure 1:**
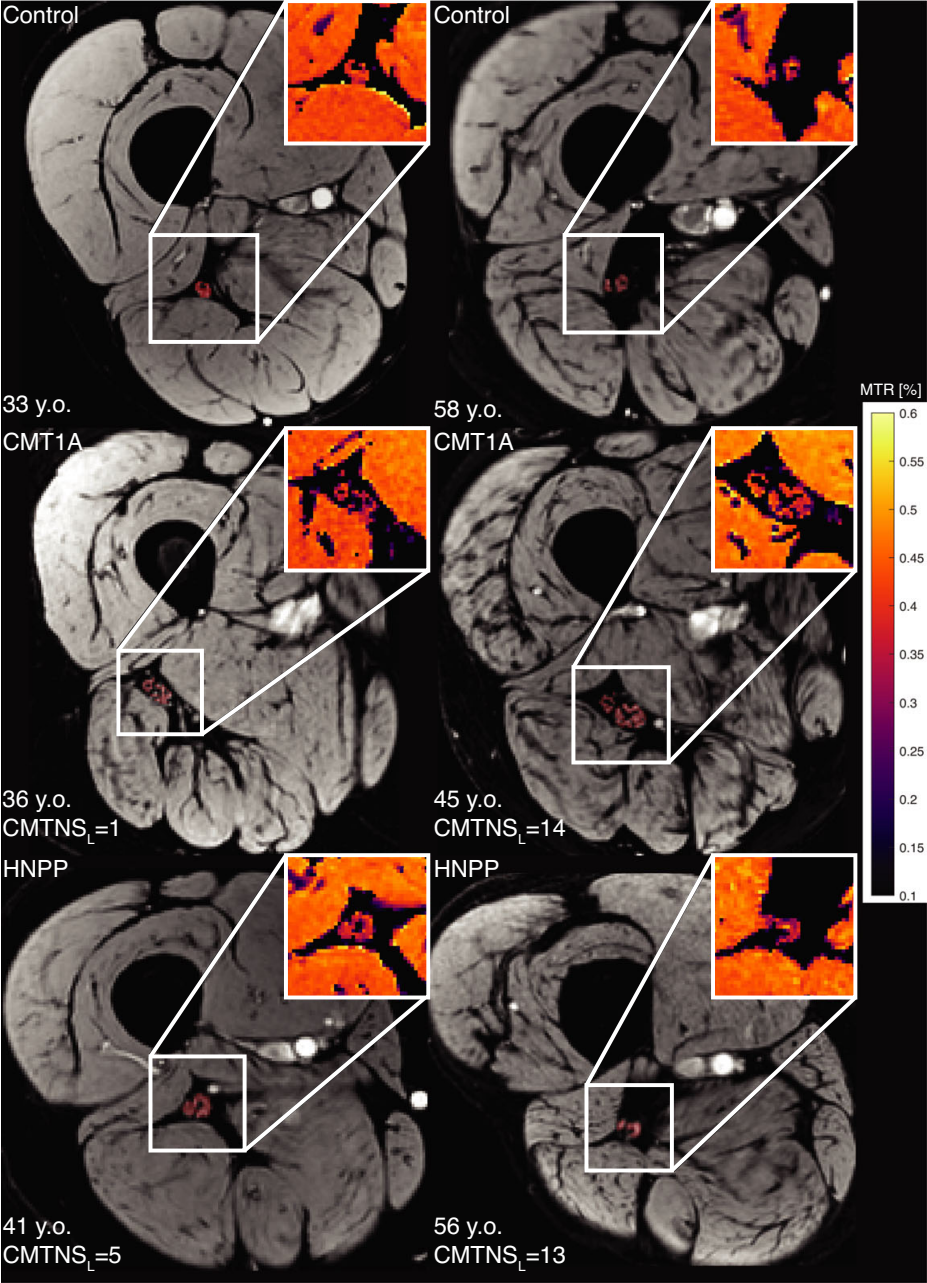
Representative anatomical images of study subjects with sciatic nerve segmentations overlaid in red. The magnified magnetization transfer ratio (MTR) maps are shown in the inset. The subjects in row 1 are healthy control subjects, row 2 have Charcot–Marie–Tooth type 1A (CMT1A), and row 3 have hereditary neuropathy with liability to pressure palsy (HNPP). The subjects in the left column represent younger individuals with lower levels of disability, while the subjects in the right column represent older individuals with higher levels of disability. Note the enlarged nerve in the CMT1A patients that increases with disability.

Scans were well tolerated in all subjects and the images were free of artifacts. Figure 1 shows sample reference anatomical images and zoomed MTR maps of the sciatic nerve in control subjects and patients. The sciatic nerve was readily distinguishable from the surrounding fat and muscle, with the fat signal being nearly fully suppressed by the water‐selective excitation pulse. Note that our imaging volume included the bifurcation of the sciatic nerve into the common peroneal and tibial nerves in the most distal slices. Both branches were included in the sciatic nerve ROI for the analyses.

### Significant differences in MTR, CSA, and circularity between CMT1A patients and control subjects

**Figure 2:**
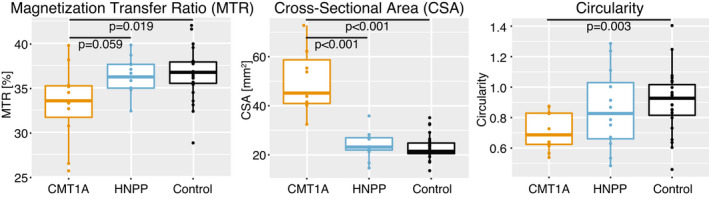
Box and whisker plots with data points of magnetization transfer ratio (MTR), sciatic nerve cross‐sectional area (CSA), and circularity by patient cohort. CMT1A, Charcot–Marie–Tooth type 1A; HNPP, hereditary neuropathy with liability to pressure palsy. Note the decreased MTR in CMT1A patients relative to matched controls. Similarly, note the increased CSA and decreased circularity of CMT1A nerves. No differences were observed for the HNPP cohort relative to controls.

Differences in MTR were observed between CMT1A patients and control subjects (*p* = 0.019) as shown in Figure 2. As can be seen in Figure 1, sciatic nerve hypertrophy was observed in CMT1A patients compared to HNPP patients and control subjects. Quantitatively, CMT1A patients had significantly larger CSA compared to control subjects (*p* < 0.001, Fig. 2). Finally, CMT1A patients had significantly lower circularity (less cylindrical sciatic nerves) compared to control subjects (*p* = 0.003). No significant differences in MTR, CSA, or circularity were found between the HNPP patients and control subjects.

### MTR and CSA correlate to clinical metrics

**Figure 3:**
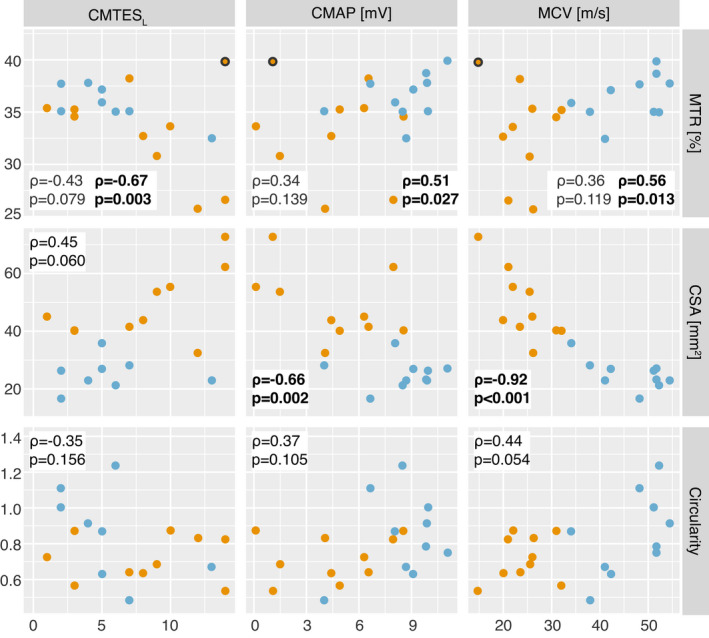
Scatterplot of clinical and imaging metrics with Spearman correlation coefficients (*ρ*) and *p*‐values for all patients. CMT1A patients are in orange and HNPP patients in blue. The CMT1A patient outlined in gray was found to be an outlier and was removed from MTR analysis as described in the text. The statistics in gray include the outlier patient in the analysis, while the statistics in black are the results with the patient removed. Significant relationships are indicated via bolded text. CMTES_L_, Charcot–Marie–Tooth Evaluation Score in the Legs; CMAP, compound motor action potential; MCV, motor conduction velocity; MTR, magnetization transfer ratio; CSA, cross‐sectional area of the sciatic nerve; CMT1A, Charcot–Marie–Tooth type 1A; HNPP, hereditary neuropathy with liability to pressure palsy.

For MTR, one outlier (gray outlined circle in Fig. 3) was identified, which was defined as a point that lay more than 2.5 standard deviations from the expected values based on a linear trendline between MTR and CMTES_L_. No other MTR outliers were identified based on this criterion. When the outlier patient was removed from the correlation between MTR and CMTES_L_, a significant correlation was found (*ρ* = −0.67, *p* = 0.003) across all remaining patients. When the same patient was removed from other MTR analyses (correlations with CMAP and MCV), significant correlations were also found (CMAP: *ρ* = 0.51, *p* = 0.027, MCV: *ρ* = 0.56, *p* = 0.013). Across all patients, CSA correlated with CMAP (*ρ* = −0.66, *p* = 0.002) and MCV (*ρ* = −0.92, *p* < 0.001). Looking across all non‐outlier CMT1A patients alone, MTR correlated with CMTES_L_ (*ρ* = −0.83, *p* = 0.006) and CSA correlated with MCV (*ρ* = −0.78, *p* = 0.012). No imaging metrics correlated with clinical metrics in the HNPP population alone. All relationships between the clinical disability scores/clinical biomarkers and each potential imaging biomarker can be seen in Figure 3.

### Significant differences in MTR were found based on sex

Significant differences were observed in MTR according to sex (*p* = 0.013); however, this conflicts with previous reports^19^ and should be interpreted with caution. No significant differences were found in CSA (*p* = 0.56) or circularity (*p* = 0.27) based on sex. MTR, CSA, or circularity were not related to age (*p* > 0.1) or BMI (*p* > 0.1) in our cohort.

### A linear multiple regression predicted clinical disability based on sex and MTR

A one‐way analysis of variance (ANOVA) test showed that sex (*p* < 0.001) and MTR (*p* = 0.045), but not CSA (*p* = 0.758), were significant predictors of CMTES_L_. As a result, CSA was removed from the model. The resulting multiple linear regression of CMTES_L_ was based on sex (normalized intercept ± standard error: 5.05 ± 0.76, *p* < 0.001; and normalized coefficient estimate: 4.19 ± 1.49 for males, *p* = 0.014) and MTR (normalized coefficient estimate: −1.69 ± 0.74, *p* = 0.038). The regression equation for CMTES_L_ was found with an adjusted‐*R* ^2^ of 0.68 and *p* < 0.001. In this model, the same outlier patient identified in the previous section was removed.

### MTR and CSA were reliable between raters

Inter‐rater reliability statistics are given in Table 2 including the mean and standard deviation extracted by raters 1 and 2 for each potential imaging biomarker, the ICC, CV, and paired *t*‐test *p*‐value across scans. MTR (ICC >0.99, CV = 0.30%) had the highest inter‐rater reliability followed by CSA (ICC = 0.99, CV = 7.25%), while circularity was less reliable (ICC = 0.65, CV = 17.65%).

**Table 2:** Inter‐rater reliability statistics.

	MTR	CSA	Circularity
Rater 1 mean ± standard deviation	33.2 ± 3.6	30.8 ± 17.0	0.88 ± 0.22
Rater 2 mean ± standard deviation	33.2 ± 3.7	32.0 ± 16.7	0.78 ± 0.18
ICC	>0.99	0.99	0.65
CV [%]	0.30	7.25	17.65
Paired *t*‐test *p*‐value	0.15	0.14	0.08

The mean and standard deviation for each metric are listed per rater along with the intraclass correlation coefficient (ICC), coefficient of variation (CV), and paired *t*‐test *p*‐value. Reliable metrics have a large ICC, small CV, and non‐significant *p*‐value (*p* > 0.05). MTR, magnetization transfer ratio; CSA, cross‐sectional area; ICC, intraclass correlation coefficient; CV, coefficient of variation.

### MTR was the most repeatable metric in control subjects

**Figure 4:**
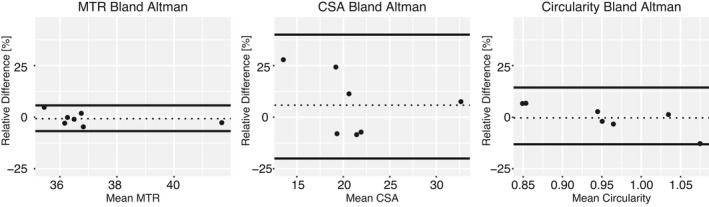
Scan–rescan Bland–Altman plots representing the 95% limits of agreement according to the relative difference in MTR, CSA, and circularity. The 95% limits of agreement were calculated based on relative difference in metrics between scans in control subjects. The dotted line is the bias. MTR, magnetization transfer ratio; CSA, cross‐sectional area.

The test–retest limits of agreement, ICC, and CV are listed for each potential imaging biomarker in Table 3. The Bland–Altman plots are shown for all imaging metrics in Figure 4. MTR had the narrowest 95% limits of agreement (−6.75%, 5.71%) followed by circularity (−13.12%, 14.26%). CSA had the widest limits of agreement (−20.01%, 39.99%).

**Table 3:** Test–retest repeatability statistics.

Metric	MTR	CSA	Circularity
LOA (lower, upper) [%]	(−6.75, 5.71)	(−20.01, 39.99)	(−13.12, 14.36)
ICC	0.84	0.89	0.55
CV [%]	3.16	13.01	7.10

Metrics with high repeatability have narrow 95% LOA with a large ICC and small CV. MTR, magnetization transfer ratio; CSA, cross‐sectional area; LOA, limits of agreement; ICC, intraclass correlation coefficient; CV, coefficient of variation.

## Discussion

This study evaluated multiple imaging metrics related to the sciatic nerve microstructure and macrostructure for their suitability as monitoring biomarkers in future clinical trials of CMT1A and HNPP. The sensitive and reliable biomarkers evaluated herein may provide much needed information on disease progression and treatment response that are critically needed for future drug development trials, although additional longitudinal studies are needed to evaluate their sensitivity to change over time. This will be particularly important in CMT1A and HNPP because of their slowly progressive natures and the fact that treatments are more likely to slow or stop progression rather than reverse it. Note that these metrics were not developed for diagnostic purposes, as genetic testing is usually sufficient to establish the diagnosis.

This study revealed significant differences in MTR (*p* = 0.019), CSA (*p* < 0.001), and circularity (*p* = 0.003) between CMT1A patients and control subjects (Fig. 2). The significantly lower MTR observed in CMT1A patients likely represents a decrease in myelin content from chronic nerve degenerative processes and secondary axonal loss in CMT1A. The increase in sciatic nerve CSA and decrease in circularity (less cylindrical) likely stem from proliferation of endo/peri/epineurial tissues and excessive collagen fiber deposition in CMT1A.^32^ The results herein are consistent with previous imaging studies, which observed CMT1A nerve hypertrophy compared to control subjects or other CMT subtypes.^20^, ^33^, ^34^, ^35^, ^36^ In our current study, there were no significant differences between HNPP patients and healthy control subjects, which may conflict with a previous study that found a trend toward reduced sciatic nerve MTR values in another HNPP cohort.^9^ In addition, future work focused on measurements in both proximal and distal nerves to evaluate the length‐dependence of these features is warranted, as length‐dependence has been shown previously in the intramuscular fat percentages measured on MRI in HNPP patients.^9^

Significant correlations were observed between imaging metrics and clinical metrics in the CMT1A and all patient populations (CMT1A: MTR with CMTES_L_ (*p* = 0.006) and CSA with MCV (*p* = 0.012), all patients: MTR with CMTES_L_ (*p* = 0.003), CSA with CMAP (*p* = 0.002), and CSA with MCV (*p* < 0.001, Fig. 3). The observed correlation between MTR and clinical biomarkers was consistent with previous reports and the idea that de/dysmyelination play a significant role in the pathogenesis of both neuropathies.^19^ A previous animal study found that MT contrast was significantly elevated in *PMP22* animal models with heterozygous deletions of *PMP22*, which may be reflective of increased myelination from tomaculae formation.^37^ In contrast, MT contrast was significantly reduced in age matched CMT1A (C3 model) nerves, which may reflect both myelin and axonal loss. In other words, HNPP nerves may exhibit different baseline levels of myelination relative to both healthy and CMT1A nerves, making comparisons across cohorts difficult. However, MTR may still provide insight into progressive myelin and axonal loss, which will be the focus of future longitudinal studies in both HNPP and CMT1A patients.

A multiple linear regression of the proposed imaging metrics (MTR, CSA) and sex (significant relationship with MTR according to Wilcoxon rank‐sum test in controls) predicted CMTES_L_. The model's most predictive variable was sex, where males had more severe disability compared to females, contradicting a previous study by Padua et al.^38^ Rather than relying on problematic feature selection methods,^39^ MTR and CSA were manually selected, as they had previously shown promise as biomarkers.^9^, ^19^ CSA was then removed after ANOVA showed that including CSA added no value. These results, however, should be interpreted cautiously, given the small number of patients (*N* = 18 across both cohorts). Ideally, a model would be built for each disease type or a single model with disease‐specific interaction terms. However, given our patient size, this was not possible. Nevertheless, the results herein demonstrate that MTR is an important predictor of disability and demographic factors (sex) must be considered as covariates in future studies.

Next, the performance characteristics (inter‐rater reliability and inter‐scan repeatability) of each candidate imaging biomarker were evaluated. This is a critical step in biomarker development, as it dictates the minimum change needed to reliably detect a true biological change due to progression or treatment response. Of the metrics evaluated, MTR had the highest inter‐rater reliability (Table 2). A previous study investigating the inter‐rater reliability of MTR in the sciatic nerve in healthy controls found the ICC to be 0.81, lower than the ICC found in our study (ICC >0.99). Two other studies with smaller subject numbers also investigated the inter‐rater reliability with ICC: ICC = 0.65 in five subjects by Yiannakas et al. and ICC >0.99 by Dortch et al. in 13 subjects.^19^, ^40^ CSA and circularity had higher CVs across raters, likely due to the sensitivity of these metrics to the manually selected ROI rather than the mean image intensity within the ROI. We postulate that inter‐rater and intra‐rater reliability (not assessed here) may be improved with the implementation of automated or semi‐automated segmentation algorithms.

Finally, the inter‐scan repeatability was established for each imaging metric. Test–retest limits of agreement were established based on two scans performed in seven healthy control subjects (Table 3, Fig. 4). Despite the long time between scans and small number of patients with two scans, narrow limits of agreement, with a high ICC and low CV were found for MTR. A previous study of the repeatability of MTR in the sciatic nerve by Preisner et al. found lower test–retest ICC values (0.75–0.79)^41^ than those found in our study (0.84). Two studies with smaller patient sizes also reported test–retest repeatability ICCs: Dortch et al. reported an ICC of 0.92 in the sciatic nerves of *N* = 13 control subjects and Yiannakas et al. 0.76 in the proximal lumbar plexus of *N* = 5 control subjects.^19^, ^40^ Yiannakas also reported a CV in the proximal lumbar plexus of 3.2%, which closely agrees with our findings (CV = 3.16%). The higher ICC values observed both in this study and the previous Dortch et al. study compared to that by Preisner et al. may be due to the differences in scan volumes and samples sizes. The wider limits of agreement and larger CVs found for CSA and circularity likely reflect issues related to manual ROI selection as described above for inter‐rater reliability. Although we attempted to correct for the effect of oblique slices on CSA estimates, this was not possible for the circularity estimates and may be an additional source of variance across scans. Nevertheless, the proposed imaging biomarkers herein demonstrated promising levels of test‐retest reliabiltiy, but the reported test–retest statistics should be interpreted with caution given the small sample size and previous studies indicating lower CVs and wider limits of agreement.

One limitation of this study was the small sample size and incomplete data collected for the clinical biomarkers (e.g., NCS and disability scores were not available in all patients and none of the control subjects). In addition, the imaging and clinical data were not always acquired on the same day. This is expected to have a larger impact in the HNPP patients given the transient nature of the disease and may partially account for the limited relationship observed between imaging and clinical findings. One further limitation is that the NCS was performed in the arms while the imaging in the legs, which may reduce the observed relationship between imaging and NCS findings. This was done because a limited NCS response is expected in the legs of these patients. However, because polyneuropathies are symmetric and length‐dependent, nerve conduction in forearms and MRI in legs are both expected to relate to disease progression. In addition, future work is needed to compare these nerve biomarkers to downstream biomarkers in muscle, as previous studies have investigated the replacement of muscle with fat in these patients.^9^, ^18^ Future work should also include separate investigations into patients with other polyneuropathies (e.g., chronic inflammatory demyelinating polyradiculoneuropathy), as we show here that nerve imaging biomarkers may not generalize across neuropathies (e.g., nerve hypertrophy was unique to CMT1A in our cohort of CMT1A and HNPP patients).

Despite these limitations, this study demonstrated that MTR, CSA, and circularity show promise as biomarkers in CMT1A. Data were less clear in the HNPP patient population, possibly due to its transient nature and the effect of different baseline myelin content levels across cohorts due to tomacula. Together, these findings suggest that sciatic nerve MTR and CSA estimates are sensitive, reliable, and repeatable measures of CMT1A pathology, and additional longitudinal studies are warranted to evaluate their responsiveness to disease progression and treatment response in future human multi‐center trials.

## Conflict of Interest

No authors in this manuscript have any conflict of interest to disclose.

## Author Contributions

A. R. R.: analysis and interpretation of data, statistical analysis, and drafting/revising the manuscript, J. L.: study concept and design, acquisition of data, funding support, drafting/revising the manuscript, and interpretation of data. R. D. D: study concept and design, acquisition of data, analysis and interpretation of data, statistical analysis, funding support, study supervision, and drafting/revising the manuscript.
